# Molecular Characterization of AEBP1 at Transcriptional Level in Glioma

**DOI:** 10.1155/2021/5579359

**Published:** 2021-07-31

**Authors:** Kuanyu Wang, Ruoyu Huang, Xuezhi Tong, Zhiliang Wang, Shibin Sun, Chenxing Wu

**Affiliations:** ^1^Department of Gamma Knife Center, Beijing Neurosurgical Institute, Capital Medical University, Beijing, China; ^2^Department of Neurosurgery, Beijing Neurosurgical Institute, Capital Medical University, Beijing, China; ^3^Department of Neurosurgery, Sanbo Brain Hospital, Capital Medical University, Beijing, China

## Abstract

**Background:**

Glioma is the most common malignant tumor of the brain in adult patients. The standardized treatment protocol is based on surgical therapy, supplemented with radiotherapy and chemotherapy. However, the prognosis is still unsatisfied. Chemoresistance is one of the most important reason for the poor prognosis of glioma patients. It has confirmed that glioma stem cell (GSC) is one of the reasons for chemoresistance.

**Methods:**

In this study, three datasets (GSE23806, COSMIC, and TCGA) were used to perform the analysis to search for the key genes related to GSC, temozolomide (TMZ) resistance, and prognosis. The key gene for further research was selected by reviewing the previous studies. The selected gene investigated the relation between expression levels and clinical characteristics in both TCGA and CGGA dataset. The bioinformatics analysis was performed by Gene Ontology (GO) analysis. The survival analysis was performed by Kaplan–Meier survival analysis.

**Results:**

AE binding protein 1 (AEBP1) was selected for further analysis. AEBP1 was overexpressed in GSCs and TMZ resistance cells. In both TCGA and CGGA dataset, the results showed that the expression level of AEBP1 was increased in glioblastoma (GBM) samples, IDH wild-type samples, and MGMT promoter unmethylated samples. Meanwhile, AEBP1 expression was positively related to several GSC markers. GO analysis showed that AEBP1 was related to immune response, cell adhesion, apoptotic process, inflammatory response, positive regulation of cell proliferation, angiogenesis, response to drug, and response to hypoxia. The survival analysis showed that the overexpressed level of AEBP1 was correlated with short survival time in both glioma and GBM patients.

**Conclusion:**

In summary, AEBP1 was related with GSC-induced TMZ resistance. Our study showed that AEBP1 might be an oncogene and a new effective therapeutic target for the treatment of glioma.

## 1. Introduction

Glioma is the most prevalent type of primary brain tumor in adults which is devastated to patients [1]. According to the 2016 World Health Organization (WHO) CNS tumors classification, glioblastoma multiforme (GBM) is the most malignant type of glioma with a median survival of 14.4 months [2, 3]. Despite the therapies including surgery, chemotherapy and radiotherapy are improved, and the treatment effects are still unsatisfied especially in GBM patients. New effective treatments are urgently needed to further improve the treatment effect.

The standardized drug used in chemotherapy for glioma is temozolomide (TMZ) which has been defined to improve the clinical outcomes when used alone or in combination with radiotherapy. However, the effect often lasts a short time when patients suffered chemoresistance. The mechanism of chemical resistance remains unclear. Several researches have focused on the glioma stem cells (GSC) which may be associated with chemoresistance [4].

Glioma stem cells (GSCs) are designated as a subpopulation of tumor cells which show a similar characterization of neural stem cells [5]. It has been defined that GSCs have the characterization of self-renewal, long-term proliferation, and multilineage differentiation potential in several researches [6–8]. GSCs may survive during chemotherapy and differentiate to tumor cells which proliferate rapidly. It could lead to chemoresistance and tumor recurrences [9]. In recent years, several genes have been reported as signatures of GSCs. CD133 (PROM1) is one of the most reported genes as a GSC biomarker [10]. It has been reported that glioma cells with CD133 positive can grow spheres in serum-free medium, whereas CD133 negative glioma cells cannot grow [7, 11]. It has been demonstrated that the tumorigenicity could weaken when CD133 was knocked down [11]. SRY-box transcription factor 2 (SOX2) is another signature of GSCs which overexpressed in GSCs. The glioma cells could stop proliferation and loss tumorigenicity when SOX2 was silenced [12].

However, the researches for GSC-related genes to predict prognosis and chemoresistance are insufficient. Under these circumstances, we focused on these GSC-related genes to search for a new biomarker in glioma which was also related to chemoresistance. In the present study, we discovered that the mRNA expression of AE binding protein 1 (AEBP1) was overexpressed in TMZ chemoresistance cell lines and glioma stem cell lines. AEBP1 was a coding protein which encoded a member of carboxypeptidase A protein family. In the previous researches, it was defined that AEBP1 was overexpressed in GBM and related with many types of tumor such as colon adenocarcinoma and gastric cancer [13–15]. So we focused on AEBP1 to research for its molecular and clinical characterization in glioma.

## 2. Methods

### 2.1. Samples

In this research, two datasets were used to select the target gene which related to the GSCs and chemoresistance. GSE23806 dataset contained RNA microarray data of glioma stem cell lines and conventional glioma cell lines which was downloaded from the public website (https://www.ncbi.nlm.nih.gov/geo/). The COSMIC dataset contained the half-maximal inhibitory concentration (IC50) to TMZ and the RNA microarray data of several glioma cell lines which were obtained from the public website (https://cancer.sanger.ac.uk/cell_lines/). Another two datasets were used to do the analysis of the AEBP1 characterization. The Cancer Genome Atlas (TCGA) RNA sequencing dataset was obtained from the website (http://cancergenome.nih.gov) which contained 699 glioma samples. The Chinese Genome Atlas (CGGA) RNA sequencing dataset was obtained from the website (http://www.cgga.org.cn) which contained 693 glioma samples. The expression value of the RNA sequencing data was log-transformed before analysis. Thus, in total, 1392 samples were included in this research.

### 2.2. Bioinformatics Analysis

Gene Ontology (GO) analysis was used to do the biological function analysis [16]. R programming language was used to analyze the correlation between AEBP1 expression and other genes expression with the Pearson correlation analysis. The positive-related genes were selected to do the GO analysis by DAVID (https://david.ncifcrf.gov/) [17]. The figures were plotted by the R programming language.

### 2.3. Statistical Analysis

In this study, R programming language software was used to perform the analysis and draw the figures. The analysis of the different expression levels of AEBP1 between different grades, IDH status, and MGMT promoter status was performed by Student's *t*-test. The survival analysis was performed by Kaplan–Meier (K-M) survival analysis. The circled figure was analyzed and plotted by the R programming language software [18].

## 3. Results

### 3.1. Gene Selection

**Figure 1:**
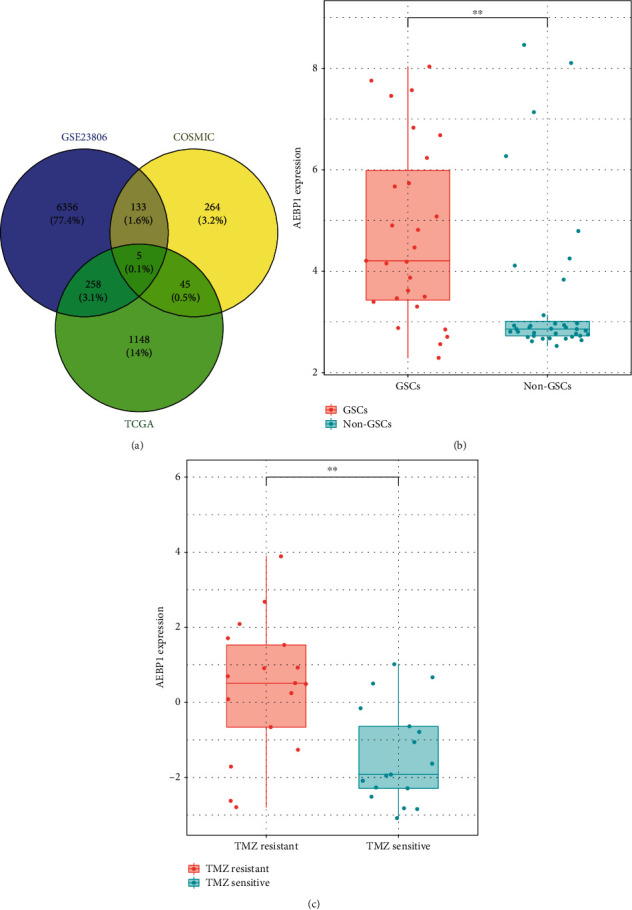
(a) The gene selection process. (b) AEBP1 expression between GSCs and conventional GBM cell lines. (c) AEBP1 expression between TMZ-sensitive cell lines and TMZ-resistant cell lines. ^∗∗^*P* < 0.01.

To search for the key gene related to with GSCs and chemoresistance, the GSE23806 dataset and the COSMIC dataset were analyzed first. In the GSE23806 dataset, the different expression genes between glioblastoma stem-like cell lines (*n* = 27) and conventional glioma cell lines (*n* = 36) were carried out by the Student's *t*-test. The genes which showed high expression in glioblastoma stem-like cell lines (*n* = 6752) were selected for further analysis. In the COSMIC dataset, the IC50 of TMZ was given, according to the median IC50, and the samples were divided into two groups: TMZ-sensitive group and TMZ-resistant group. The Student's *t*-test was carried out to find out the different expression genes between the TMZ-sensitive group and TMZ-resistant group. The high expression genes in the TMZ-resistant group (*n* = 447) were selected for further analysis. In the TCGA RNA sequencing dataset, the genes related to malignant survival in GBM patients which were carried out by univariate Cox regression (*n* = 1456) were selected for the next analysis. Only five genes were in all the three groups of the analysis result including AEBP1 which had been reported overexpressed in GBM (Figure 1(a)). The other four genes were EFEMP2, GPX2, NUDT9, and PTPRN2. As it was showed in the figures, the RNA expression level of AEBP1 was higher in GSCs and TMZ resistance cell lines than the non-GSCs and TMZ-sensitive cell lines (Figures 1(b) and 1(c)).

### 3.2. AEBP1 Expression Characterization in Glioma

**Figure 2:**
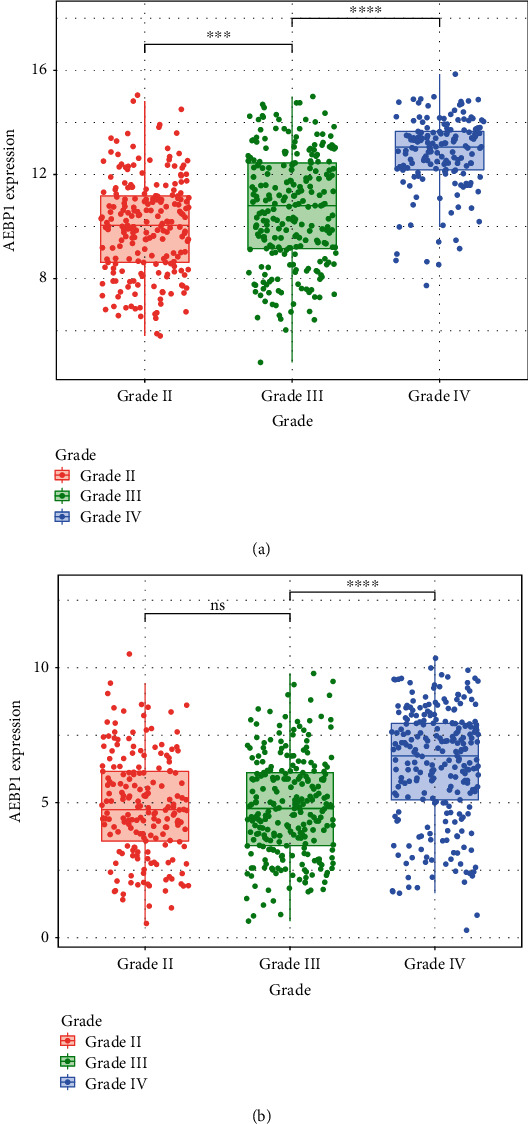
(a) AEBP1 expression with different grades in TCGA dataset. (b) AEBP1 expression with different grades in CGGA dataset. ^∗∗∗^*P* < 0.001.

**Figure 3:**
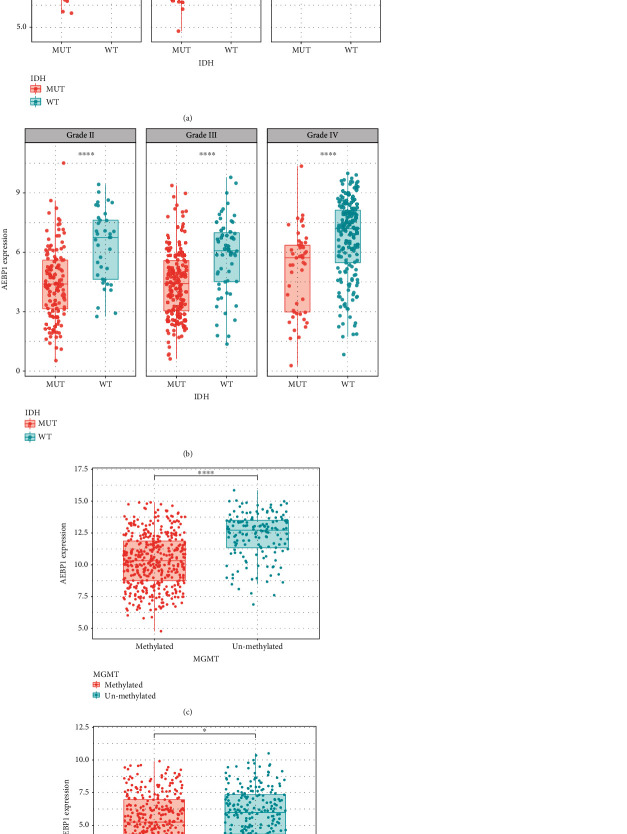
(a) AEBP1 expression with different IDH status in TCGA dataset. (b) AEBP1 expression with different IDH status in CGGA dataset. (c) AEBP1 expression with different MGMT status in TCGA dataset. (d) AEBP1 expression with different MGMT status in CGGA dataset. ^∗∗∗^*P* < 0.001, ^∗∗^*P* < 0.01, ^∗^*P* < 0.05.

During the analysis, two datasets (TCGA RNA sequencing dataset and CGGA RNA sequencing dataset) including 1392 samples were used for the analysis. The expression level of AEBP1 during different grades of glioma was analyzed in two datasets, and the results showed that it was higher in GBM patients than that in lower-grade glioma patients (Figures 2(a) and 2(b)). This result implied that the overexpression of AEBP1 was related to higher malignancy. According to the previous reports, isocitrate dehydrogenase (IDH) mutation was related to the overall survival and could be used as a subclassifier. So the relationship between IDH mutation and AEBP1 expression was detected in the datasets. As a result, it was shown that the higher AEBP1 expression was enriched in IDH wild type glioma, which indicated the possibility of AEBP1 to be used as a predictive factor (Figures 3(a) and 3(b)). The relation between MGMT promoter methylation and AEBP1 expression was also analyzed, and the result showed that the expression of AEBP1 was higher in MGMT promoter unmethylated glioma patients (Figures 3(c) and 3(d)).

### 3.3. AEBP1 Was a Potential Marker for GSC

**Figure 4:**
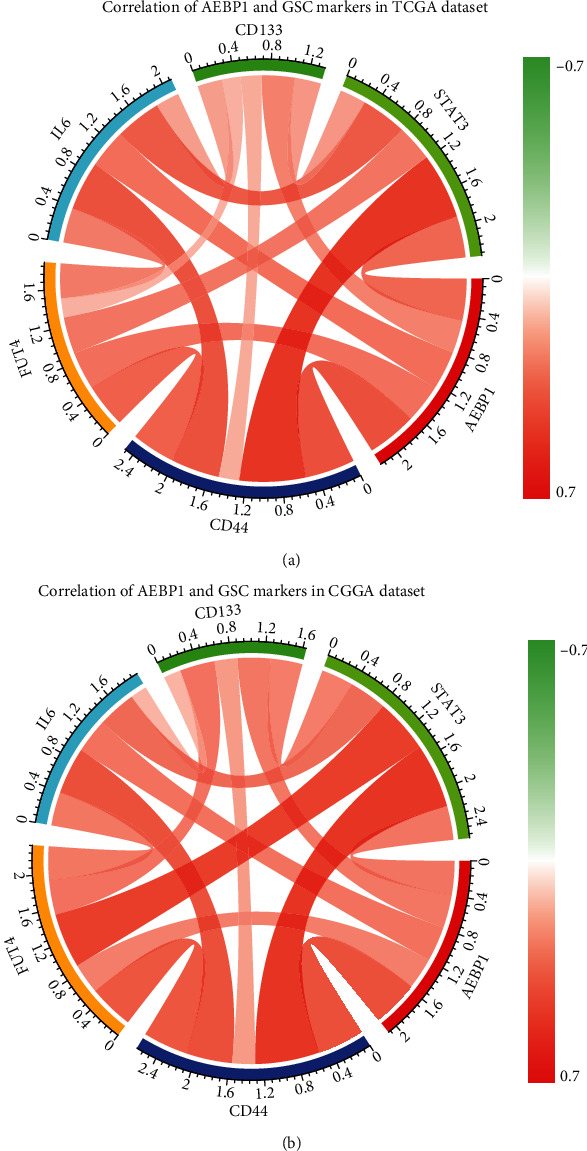
(a and b) The relation between AEBP1 expression and GSC markers expression in TCGA and CGGA datasets.

During our research, it was found that AEBP1 was upregulated in GSCs. Thus, we chose several GSC-related genes according to previous articles [19] to get further research of the relation between AEBP1 and GSC markers. The relation between AEBP1 and GSC markers was analyzed by Pearson correlation analysis in the two datasets, and the results showed that the expression of several GSC markers was positively related to AEBP1 expression, such as CD133, CD44, FUT4, IL6, and STAT3 (Figures 4(a) and 4(b)). IL6/STAT3 pathway had been demonstrated that played a contributing factor for multidrug resistance in cancer [20]. This result demonstrated that AEBP1 might play an important role in GSCs and lead to tumorigenicity and chemoresistance.

### 3.4. AEBP1-Related Biological Process

**Figure 5:**
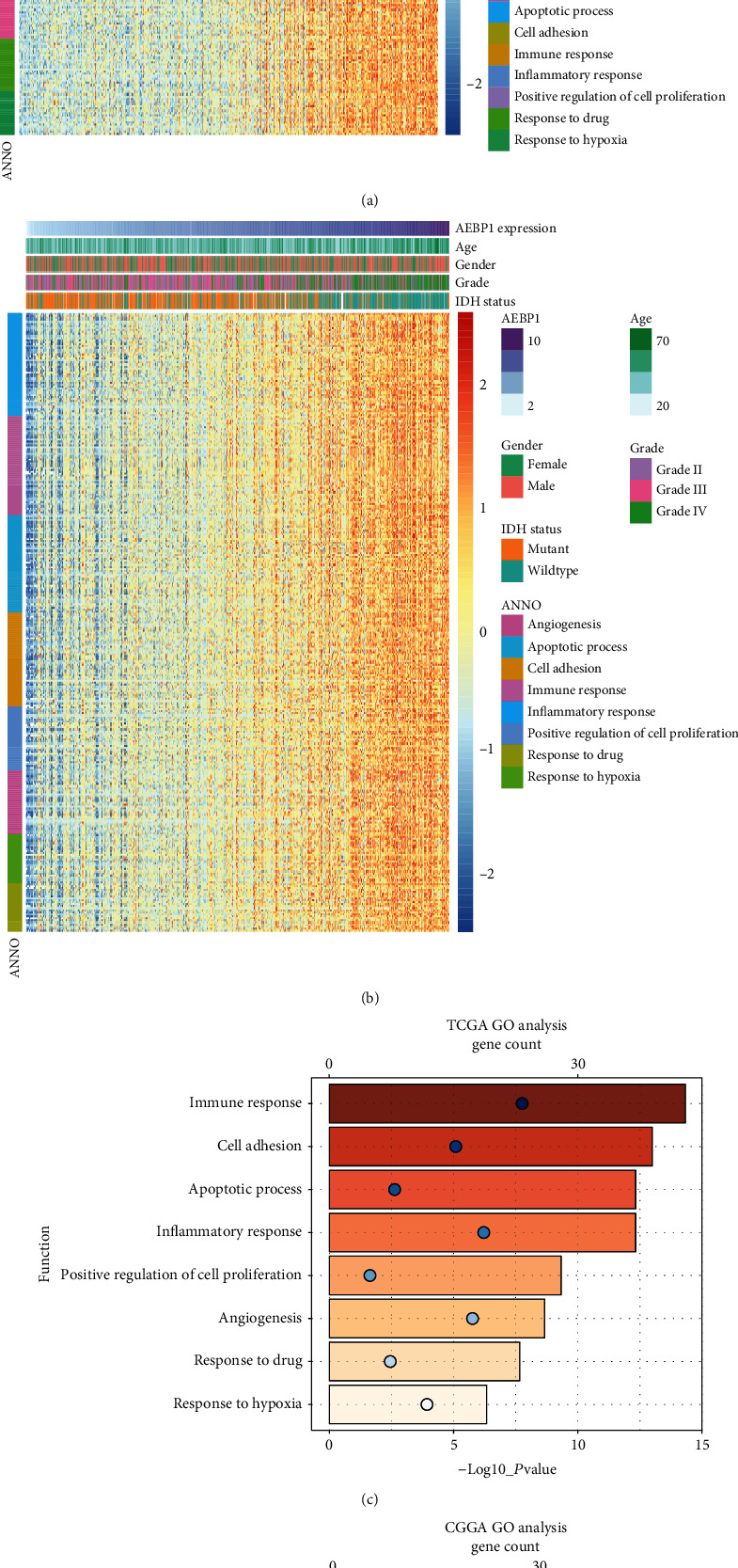
(a and c) Gene Ontology analysis of AEBP1 in TCGA dataset. (b and d) Gene Ontology analysis of AEBP1 in CGGA dataset. The samples were ranked according to AEBP1 expression from low to high.

**Figure 6:**
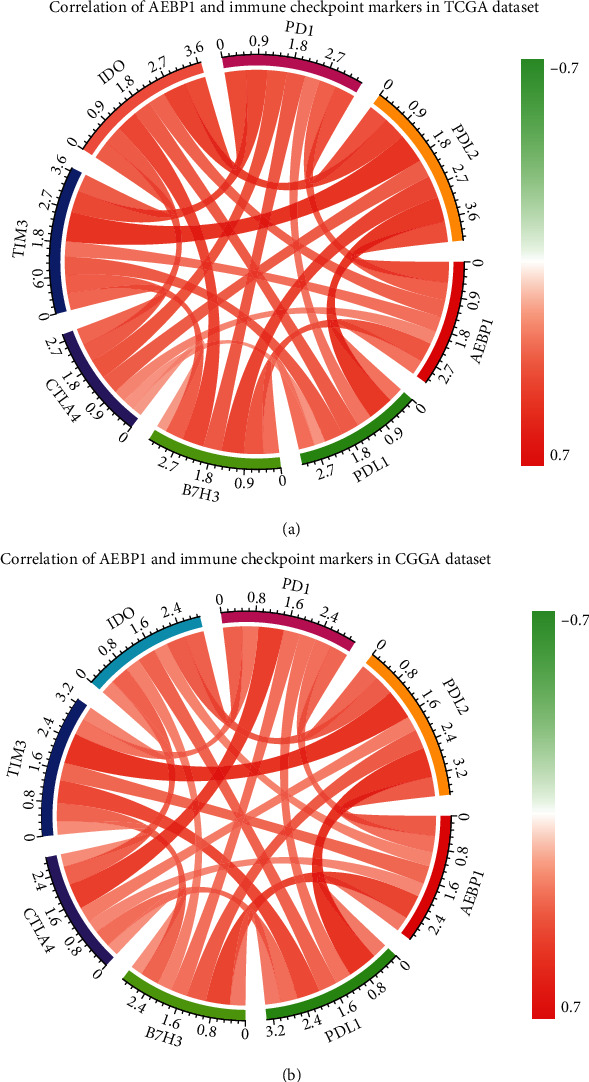
(a and b) The relation between AEBP1 expression and immune checkpoint markers expression in TCGA and CGGA datasets.

**Figure 7:**
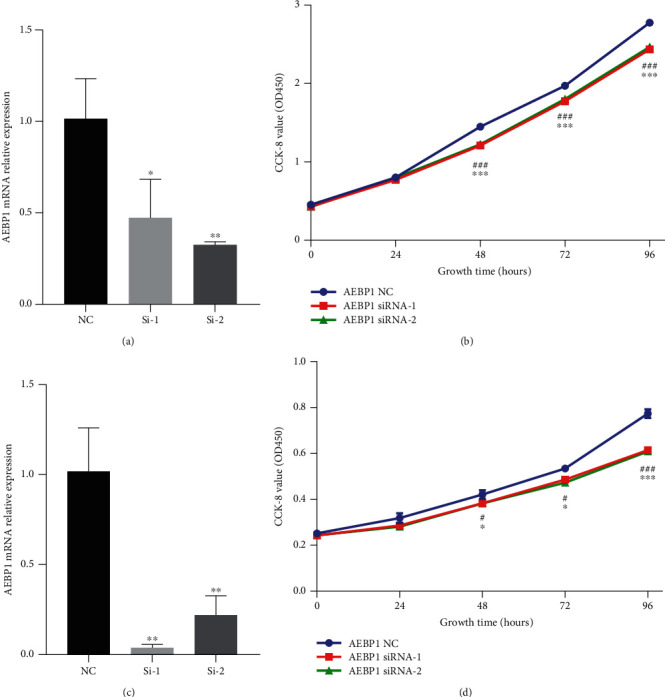
(a and c) AEBP1 mRNA expression was downregulated by siRNA in U87 (a) and GSC20 (c). (b and d) Results of cell proliferation experiment indicated that knockdown of AEBP1 expression suppressed glioma cell proliferation in U87 (b) and GSC20 (d).

Based on the above discoveries, it was supposed that AEBP1 might play an important role in the biological process of glioma. To investigate the potential function of AEBP1 in glioma, Gene Ontology (GO) analysis was performed in the datasets. The positive-related genes were found out by Pearson correlation analysis which *R* > 0.5 and analyzed with DAVID. As the results showed, the positive-related genes with AEBP1 were highly enriched in immune response, cell adhesion, apoptotic process, inflammatory response, positive regulation of cell proliferation, angiogenesis, response to drug, and response to hypoxia in GO terms (Figures 5(a) and 5(d)). The relation between AEBP1 and immune checkpoint markers [21] was also analyzed, and the results showed that the expression of immune checkpoint markers was positively related with AEBP1 expression (Figures 6(a) and 6(b)). All the results showed above indicated that AEBP1 might play an important role in the malignant progression and chemoresistance in glioma. The vitro experiments demonstrated that silencing AEBP1 expression suppressed the proliferation of glioma cells (Figures 7(a)–7(d)).

### 3.5. AEBP1 Was a Prognostic Factor for GBM Patients

**Figure 8:**
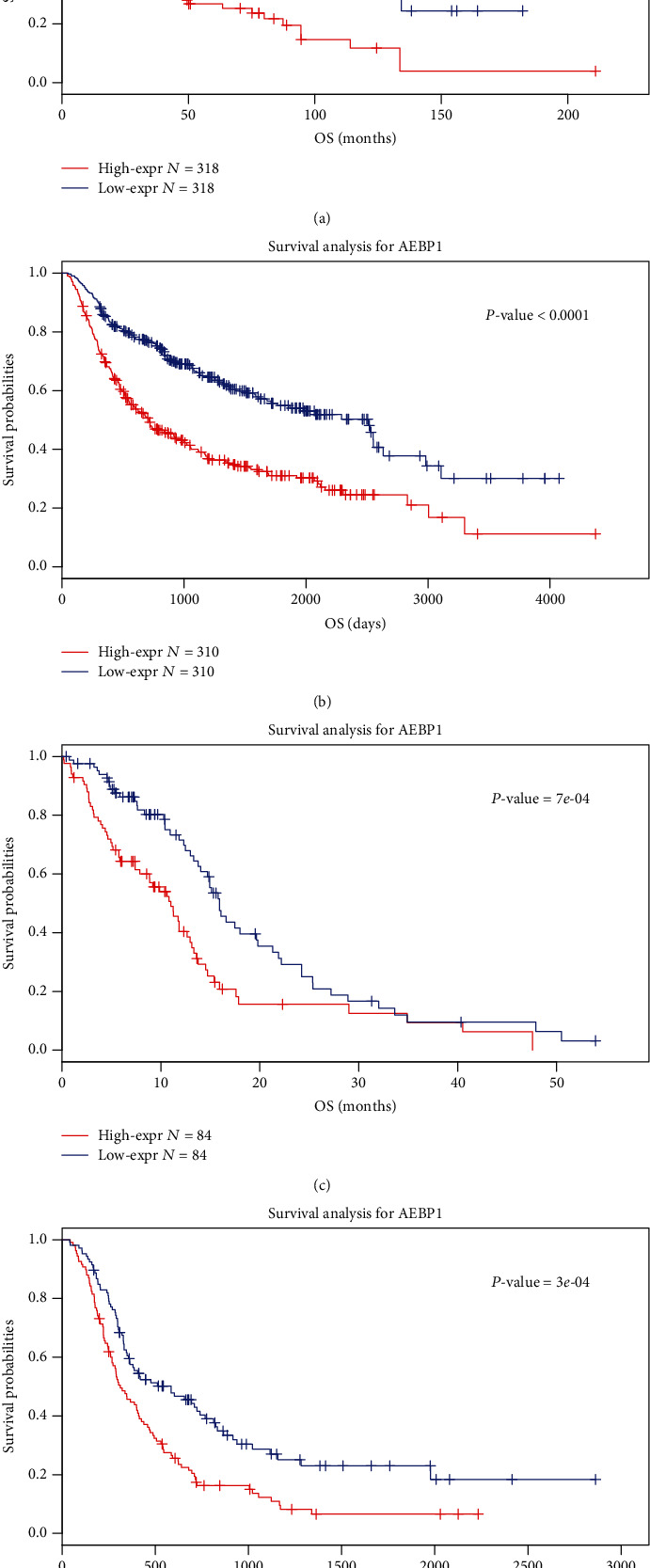
(a and b) Survival analysis of glioma samples in TCGA and CGGA datasets. (c and d) Survival analysis of GBM samples in TCGA and CGGA datasets.

To evaluate the influence of survival, we tested the prognostic value of AEBP1 expression in the datasets. Kaplan–Meier (K-M) survival curve analysis was used for the analysis. The results showed that glioma patients with higher AEBP1 expression have a shorter overall survival (OS) time (Figures 8(a) and 8(b)). Because of the heterogeneity between different grades of glioma, we analyzed the prognostic value of AEBP1 in GBM patients additionally. Similar results were obtained in GBM patients that patients with higher AEBP1 expression had a shorter OS time (Figures 8(c) and 8(d)). These results demonstrated that AEBP1 might be an effective prognostic biomarker for glioma patients, especially GBM patients.

## 4. Discussion

Glioma is the most common primary malignant tumor in the central nervous system. Even the treatment is advanced, and the median survival time of GBM patient is still unsatisfied. The origin and development of glioma are complex processes with multiple factors and steps. Following the advent of second-generation sequencing technology, the abnormal metabolic reorganization and significant cellular and molecular heterogeneity have detected in glioma cells which explains the poor conventional treatment effects, easy tumor recurrence after treatment, and difficulty in the availability of effective and innovative treatments. TMZ is the first-line chemotherapeutic drug for malignant glioma. The effective rate of TMZ in treating glioma is about 45%. The main reason for the failure of chemotherapy is the resistance of TMZ. Several studies have found that TMZ resistance in glioma is a combination of multiple factors, including DNA damage repair, glioma stem cell, and the expression of oncogenes and tumor suppressor genes in tumor cells.

In this study, we focused on TMZ resistance which caused by GSCs. By analyzing the COSMIC and GSE23806 datasets, we found that the mRNA expression level of AEBP1 was upregulated in TMZ-resistant cell lines and glioma stem cell lines. AEBP1 encoded a member of the carboxypeptidase A protein family located on Chr7. AEBP1 was widely present in fat, liver, brain, and lung tissues, but rarely detected in blood [22]. The encoded protein might act as a transcriptional repressor and play an important role in adipogenesis and smooth muscle cell differentiation. In addition, some studies have found that the expression level of AEBP1 in the proliferative precursor adipocytes was higher, while the terminally differentiated nonproliferative adipocytes disappeared [23].

Further analysis was performed in TCGA and CGGA datasets, and the results showed that AEBP1 expression in GBM patients was significantly higher than that in lower-grade glioma patients. It was also found that AEBP1 expression was overexpressed in IDH wild type glioma patients and MGMT promoter unmethylation-type glioma patients. In many previous researches, it had been demonstrated that the prognosis of the IDH wild type was malignant. This result was consistent with the poor prognosis of patients with high expression of AEBP1. The status of MGMT promoter was demonstrated as a factor to predict TMZ sensitivity, and the MGMT promoter unmethylation type glioma was not sensitive to TMZ. AEBP1 was overexpressed in MGMT promoter unmethylation type glioma which might be an important factor in predicting TMZ sensitivity.

Several studies have confirmed that glioma stem cell was an important cause of resistance to radiation therapy and chemotherapy in glioma [24–26]. It has been reported that glioma stem cells may survive after chemotherapy and then differentiate into tumor cells and cause tumor recurrence [9]. Through our research, it was demonstrated that the expression of AEBP1 was significantly upregulated in GSCs, and the expression of multiple GSC markers was positively correlated with the expression of AEBP1, such as CD133, CD44, FUT4, IL6, and STAT3. Several studies have confirmed that the IL6/STAT3 pathway was closely related to tumorigenesis and self-renewal caused by GSCs. This result indicated that AEBP1 might play an important role in maintaining the survival of GSCs and lead to tumorigenicity and chemotherapy resistance. These results suggested that the relevant targeted therapy can be used to block the signal transduction pathways related to GSCs growth, which might be a new direction of glioma treatment.

In this study, the bioinformatics analysis was also performed. The results showed that the genes positively related to AEBP1 were enriched in functions such as immune response, cell adhesion, apoptotic process, inflammatory response, positive regulation of cell proliferation, angiogenesis, response to drug, and response to hypoxia. In these results, AEBP1 was closely related to inflammation response and immune response which might lead to immune escape of tumor cells. The immune checkpoint markers were positively related to AEBP1 expression. It indicated that the AEBP1 might suppress the immune response through certain pathways. Therefore, it was inferred that the antibody of AEBP1 might be a new select for effective treatment in glioma patients. In addition, AEBP1 was positively related to response to drug and apoptotic process, which might be one of the reasons for the high expression of AEBP1 causing resistance to TMZ chemotherapy. The positive-related genes with AEBP1 were also enriched in positive regulation of cell proliferation, angiogenesis, response to hypoxia, and cell adhesion which related with the malignant progression of glioma.

The survival analysis was performed, and the results showed that the patients with higher AEBP1 expression had a shorter overall survival time than the patients with lower AEBP1 expression. This result indicated that AEBP1 might be used as a prognostic biomarker for glioma patients. The relevant targeted therapy might be effective in glioma patients with AEBP1 overexpression.

## 5. Conclusion

In conclusion, the results described above revealed that AEBP1 might be an oncogene in glioma. It might be related to GSC and chemoresistance. The upregulation of AEBP1 might indicate poor sensitivity to TMZ therapy. The study also points to AEBP1 as a new effective therapeutic target for the treatment of glioma.

## Data Availability

All data generated and analyzed during this study are included in this published article.
